# Media coverage of Canadian Veterans, with a focus on post traumatic stress disorder and suicide

**DOI:** 10.1186/s12888-022-03954-8

**Published:** 2022-05-16

**Authors:** Rob Whitley, Anne-Marie Saucier

**Affiliations:** 1 Douglas Research Centre, Verdun, Quebec Canada; 2 Department of Psychiatry, McGill University, Montreal, Quebec Canada

**Keywords:** Veterans, Media, Suicide, Post traumatic stress disorder, Canada, Stigma, Newspaper

## Abstract

**Background:**

A large corpus of research indicates that the media plays a key role in shaping public beliefs, opinions and attitudes towards social groups. Some research from the United States indicates that military Veterans are sometimes framed in a stereotypical and stigmatizing manner, however there is a lack of research on Canadian media coverage of Veterans. As such, the overarching aim of this study is to assess the tone and content of Canadian media coverage of military Veterans, with a focus on PTSD and suicide. The first objective is to document and analyze common themes, content and temporal patterns in Canadian media coverage of Veterans per se. The second objective is to examine common themes and content in the sub-set of articles having PTSD as a theme. The third objective is to assess adherence to responsible reporting of suicide guidelines in the sub-set of articles having suicide as a theme.

**Methods:**

We used validated and systematic methods including use of key words, retrieval software and inter-rater reliability tests to collect and code news articles (*N* = 915) about Veterans from over 50 media sources during a 12-month period, with specific coding of articles about PTSD (*N* = 93) and suicide (*N* = 61).

**Results:**

Analysis revealed that the most common theme is ‘honour or commemoration of Veterans’ which occurred in over half of the articles. In contrast 14% of articles focused on danger, violence or criminality. In the sub-set of articles with PTSD as a theme, over 60% focused on danger, violence or criminality, while only around 1 in 3 focused on recovery, rehabilitation, or health/social service intervention. In the sub-set of articles about suicide, there was generally strong adherence to responsible reporting guidelines, though less than 5% gave help-seeking information. Moreover, most reporting on PTSD and suicide focused on a single anomalous murder-suicide incident, with few articles about suicide prevention, helpful resources and modifiable risk factors.

**Conclusions:**

The results reveal some encouraging findings as well as a need to diversify media coverage of Canadian Veterans. This could be achieved through targeted educational outreach to help Canadian journalists responsibly report on Veterans and their mental health issues.

**Supplementary Information:**

The online version contains supplementary material available at 10.1186/s12888-022-03954-8.

## Introduction

The Government of Canada estimates that there are around 620,000 Veterans of the Canadian Armed Forces, 88% of whom are men and 12% are women [1]. Around 25,000 of these served in World War II or the Korean war, while around 40,000 served in the recent Afghanistan conflict [1]. Considerable research indicates that these Veterans are at a higher risk of adverse mental health outcomes than the general population. For example, a large-scale survey revealed that 13% of regular force Veterans had been diagnosed with Post-Traumatic Stress Disorder (PTSD), compared to 1% of the adult Canadian population, after adjusting for age and sex [2]. This survey also found that 17% of Veterans had been diagnosed with a mood disorder such as depresion, and 11% with an anxiety disorder, which was over the double the rate of the adult Canadian population after adjusting for age and sex. Moreover, Veterans are significantly more likely to die by suicide than similarly-aged non-Veterans, with recent studies indicating that Canadian Veterans have a 1.4–1.8 higher risk of suicide than other Canadian adults when controlling for age and sex [3, 4]. Furthermore, evidence suggests that Veterans have difficulty finding secure housing, employment and communal support on leaving the military [5, 6]. This is due to a variety of factors, but some research indicates that military Veterans are sometimes stigmatized and negatively stereotyped by various sectors of society, leading to discrimination and neglect [7].

A large corpus of research indicates that the media plays a key role in shaping public beliefs, opinions and attitudes towards social groups and related societal issues, especially when the public has little social contact with the group in question [8, 9]. For example, some research indicates that the stigma associated with mental illnesses such as PTSD is fueled by sensational media coverage that often focuses on rare cases of crime and violence, with little coverage of recovery and rehabilitation [10–13]. This can contribute to a wider climate of prejudice and fear, which can lead to the social exclusion and social alienation of people with mental illnesses [8].

Some US research indicates that military Veterans are framed in a stereotypical and narrow manner in the American media. For example, one recent review paper notes that US media portrayals of Veterans commonly emphasize factors such as trauma, emotional instability and drugs/alcohol [14]. Similarly, a report from the US Substance Abuse & Mental Health Services Administration [15] notes a lack of positive well-rounded stories, instead lamenting the common use of negative and stigmatizing depictions such as ‘ticking time bombs’ or ‘damaged and potentially unstable’ when referring to American Veterans.

Furthermore, recent research indicates that Veterans are also frequently portrayed as helpless victims in news articles. One US study of two high-circulation newspapers found that 87% of all articles mentioning recent Veterans framed them as victims, with 15% specifying a mental health problem in the article [16]. This overlaps with other US research, which found that US regional newspapers frequently framed Veterans as ‘charity cases’ or ‘victims’ mistreated by society [17].

These negative stereotypes may manifest themselves most intensely in media stories about PTSD or suicide in Veterans. For example, research from various countries shows that media coverage of PTSD often focuses on crime and violence, with fewer articles on recovery or rehabilitation [18–21]. Similarly, a US study of media coverage of Veteran suicide found that 60% of articles included sensational/dramatic language, 60% attributed Veteran suicide to a single cause and 40% included suicide in the headline, while 0 % contained help-seeking information or quoted suicide experts, thus deviating from responsible reporting of suicide guidelines [22].

All this led the US National Veterans Foundation [23] to state that ‘the media is the main culprit in fostering negative stereotypes about Veterans’, with Erwin [14] similarly noting that negative coverage creates inaccurate ‘public perceptions of veterans as emotionally unstable citizens on the verge of breakdown’. Some research indicates that this can contribute to a wider climate of suspicion and fear, which can impede the reintegration of Veterans into society.

For example, one US study [24] found that Veterans reported that people often perceived them as ‘crazy’, ‘dangerous’ or ‘violent’, while another US study [25] found that hiring managers often held negative stereotypes that Veterans are ‘bitter’, ‘angry’ or ‘withdrawn’. These beliefs and associated behaviours may stem from the aforementioned negative media coverage, with a recent US experimental study finding that participants who read a short stereotypical news article about Veterans significantly increased their social distance to Veterans as a consequence [26].

The vast majority of research on media coverage of Veterans has occurred in the United States, and to our knowledge there are no published studies examining Canadian media coverage of Veterans per se, let alone with a focus on mental health issues such as PTSD or suicide. This is worrying for three reasons. First, it would be erroneous to assume that patterns of media reporting observed in the US are similar in Canada, with a recent study finding significant differences when comparing Canadian and US media reporting of Robin Williams’ suicide [27]. Second, the aforementioned research indicates that one-dimensional reporting of Veterans can perpetuate stereotypes and social distance; this can impede veteran reintegration into society, which has been identified as a policy priority in Canada [28]. Indeed, a large corpus of research indicates that media stereotyping of a group, for example people with mental illness or Veterans, can lead to increased levels of internal and external stigma [26, 29, 30]. Third, related research indicates that certain types of media reporting of suicide can lead to suicide contagion among demographically similar individuals−a phenomena known as the Werther effect [31–35].

The Werther effect has been well-documented in many nations of the world [36] including Korea [37], Austria [34, 38], the US [31, 35] and Canada [39, 40] with a recent study indicating a 16% increase in Canadian suicides in the months following the death of Robin Williams [41]. Taken together, these studies indicate that the Werther effect is associated with various media-content related factors including (i) a focus on celebrity suicides; (ii) describing the suicide method in detail, especially asphyxiation and jumping; and (iii) sensationalized reporting of suicide.

It is theorized that the Werther effect occurs because some vulnerable people may identify with the decedent and their struggles via social learning after consuming such media, which is intensified when the consumer perceives that the decedent is demographically alike (e.g. age and sex) and has a similar or elevated social status [31, 42, 43]. Such affinity, combined with sensational and detailed media coverage, may lead some vulnerable consumers to conclude that suicide is a feasible answer to ongoing struggles, leading to imitative suicidal behaviour [37, 41].

Conversely, the media can be a vector for increased awareness and education, such as pointing to available resources, highlighting stories of hope and recovery, and bringing attention to important social issues surrounding suicide [34, 40, 44–46]. Indeed, several research studies indicate a modest reduction in suicide mortality and suicidal ideation after suicide-related media reporting when the news focus has included content related to (i) mastery of crises; (ii) resilience in the face of adversity; and (iii) messages of hope and recovery [34, 39, 47, 48] This has been labelled ‘the Papageno effect’.

Interestingly, several research studies, including two large-scale Canadian studies, indicate that media coverage of murder-suicides also leads to a reduction in suicide mortality, and is not associated with any Werther effect [39, 40] This may be because susceptible individuals tend not to identify with violent ‘villains’ who ipso facto have a lowered social status and are typically presented unfavourably in the media [38].

Given this situation, the Mental Health Commission of Canada has sponsored the development and wide distribution of best practice guidelines to encourage the responsible reporting of mental health and suicide, known as Mindset, with the third edition published in 2020 [49]. These are available on a devoted website, and as a short booklet, with over 5000 distributed to journalists across Canada. Mindset includes bullet point lists of recommendations that attempt to guide the journalist to include protective information, while avoiding coverage that could lead to stigma and suicide contagion. For example, the third edition includes 16 bullet points related to writing about suicide including “do not go into details about the method used” and “do tell others considering suicide how they can get help”, while there are seven mental illness bullet points including “strive to include quotes from those affected or others like them” and “do not reinforce stereotypes (especially in headlines)”.

Several studies have examined adherence to earlier editions of Mindset reporting guidelines in relation to generic media coverage of suicide and mental illness [45, 50], but there has been little research examining Mindset in relation to Canadian Veterans. This lack of research is concerning, given the potential role of the media in influencing public stigma, as well as potentially contributing to the Werther effect and the Papageno effect.

In an attempt to address this deficit, the overarching aim of this study is to assess the tone and content of Canadian media coverage of military Veterans, with a focus on PTSD and suicide. The first objective is to document and analyze common themes, content and temporal patterns in Canadian media articles about Veterans. The second objective is to examine common themes and content in the sub-set of articles which have PTSD as a theme. The third objective is to assess adherence to Mindset guidelines in the sub-set of articles which have Veteran suicide as a theme.

## Methods

We collected and coded news media articles about Veterans over a one-year period (July 1 2020–June 30 2021). A one-year period was chosen as this time window covers the yearly cycle of events relevant to Veterans (e.g. Remembrance Day, V-E Day, Vimy Ridge Day etc.) while allowing scope to assess day-to-day coverage and random events related to Veterans over a meaningful time frame. Moreover, assessment of a one-year period is a standard procedure in exploratory studies of media coverage related to mental health issues [39, 51–53]. The July-to-July cycle also correlated with the research funding from the granting organization, as well as previous Canadian studies of media coverage of mental health issues [54].

News articles were gathered using Factiva, an extensive online news database containing a wide range of Canadian media sources [55]. This was supplemented by use of Google Advanced Search to ensure all relevant articles were being captured. Specifically, we searched 58 media sources on a daily basis during the study period. This included 47 major English-language Canadian news sources comprised of three national newspapers, six major news websites and 38 metropolitan or regional newspapers, as well as 11 French-language Canadian news sources comprised of 8 major news websites and 3 newspapers (see Additional file 1 for news sources). Specifically, all articles mentioning the term(s) ‘Veteran*’, ‘military’, ‘army’, ‘navy’ or ‘air force’ from English sources and ‘Veteran*’ or ‘ancien* combatant*’ from French sources were systematically retrieved for analysis.

This retrieval process led to a long-list of articles that needed further human screening and sorting to ensure article relevance to the study objectives. Articles were excluded if they used the word ‘Veteran’ outside of a military context (e.g. ‘a veteran politician’ or a ‘veteran hockey player’) and also if they only made a passing reference to Veterans. The rest were included for coding.

To meet the research objectives, we designed a coding schema inspired by the existing literature as well as the second edition of the Mindset guidelines, which was the most up-to-date version of the guidelines when the study began [27, 34, 46, 52]. The first section of the coding schema was applied to all included articles, assessing for the presence or absence of specific content and themes. The second section of the coding schema was applied only to the sub-set of articles that covered suicide, assessing for adherence to Mindset recommendations. We used binary coding for almost all variables in both coding sections with values defined as (1) yes-present, and (0) no- not present. The coding schema consists of the variables listed in Tables 2, 3, 4 and 5.

Article retrieval and coding was performed by the second author, who underwent training in coding and continuous supervision by the first author, an experienced researcher who has led several media research projects. This included production and utilization of a workbook that defined coding criteria for each specific item. Training also included the independent coding of a sample of included articles (*n* = 20) by both authors and an additional research assistant to assess inter-rater reliability. Kappa coefficients were calculated for each item and interpreted using Landis and Koch’s [56] guidelines. The inter-rater reliability scores between the three coders ranged from 0.65 to 0.75 with an average coefficient of 0.68 (substantial) across items. The coders met after this exercise to discuss incongruities and solutions, leading to increased agreement among raters. The last author entered all codes into Excel for storage and later analysis including production of frequency counts and proportions.

## Results

**Fig. 1:**
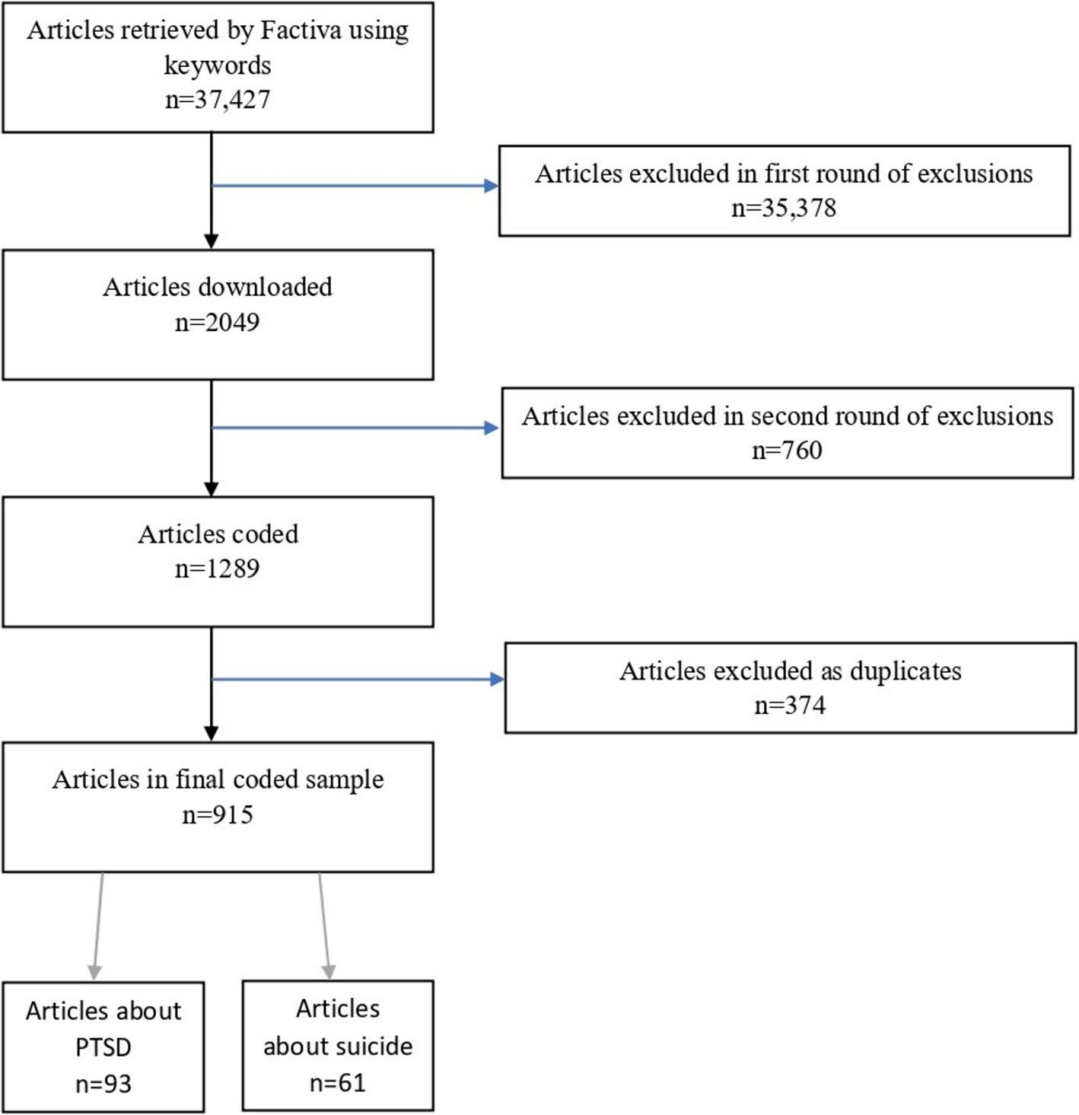
Inclusion chart depicting the filtering of articles, from retrieval to inclusion

A total of 37,427 articles were retrieved using the broad search terms over the 12-month period. As described above, this long-list was subject to further human screening and sorting to remove false positives, as well as duplicate articles. These procedures led to a final total of 915 usable articles on Canadian Veterans per se. Of these, 93 had PTSD as a theme, while 61 had suicide as a theme (see Fig. 1 for breakdown).

Of note, there were several clusters of articles over the 12-month period (see Table 1). First, over half of the articles (*n* = 478) were published in the months of October and November in the weeks surrounding Remembrance Day, mainly with themes of honour or commemoration. Second, a disproportionate number of articles (*n* = 130) appeared in the months of February and March 2021. Around a third of these (*n* = 40) reported on the inquest of Lionel Desmond, a former corporal who served in the 2nd Battalion, Royal Canadian Regiment in Afghanistan. He was diagnosed with PTSD in 2011 and medically discharged from the army in 2015. On January 3rd, 2017, police found Desmond dead at home along with his wife, daughter and mother, with the authorities concluding that this was a triple murder-suicide perpetrated by Desmond. An example headline on this topic is “Lionel Desmond’s sister tells inquiry of finding crime scene after veteran killed family and himself”.

**Table 1:** Monthly count of final coded sample, July 1, 2020–June 30, 2021

	Jul	Aug	Sep	Oct	Nov	Dec	Jan	Feb	Mar	Apr	May	Jun	SUM
Articles about Veterans per se	41	34	39	109	369	35	34	68	62	50	29	45	915
Articles with PTSD as a theme	4	2	2	6	12	2	6	21	20	4	3	11	93
Articles with suicide as a theme	0	1	0	1	4	0	0	18	22	5	2	8	61

Another cluster during the months of February and March 2021 relates to allegations of sexual misconduct leveled against a number of former military members, including retired Chief of the Defence Staff General Jonathan Vance, with 25 articles devoted to this topic. An example headline on this topic is “Complainant in case of ex-chief of the defence staff Gen. Jonathan Vance said he told her he was ‘untouchable’”. Such clustering means that there are relatively few routine articles about Veterans appearing in the media in the rest of the year.

Basic topical and content information related to the 915 articles is listed in Table 2 below. As can be seen, just under half of the news articles (43%) were focused on individual Veterans such as Jonathan Vance or Lionel Desmond. Interestingly, more articles discussed Second World War Veterans (15.7%) than Afghanistan War Veterans (9.1%), despite the greater number of Afghanistan Veterans and the recency of the latter conflict. Many of the articles discussing Afghanistan did so in the context of the Lionel Desmond incident. Some other Veterans were rarely presented in the news, including Royal Canadian Navy Veterans (3.4%), Royal Canadian Air Force Veterans (4.0%) and Female Veterans (8.1%).

**Table 2:** Basic characteristics of articles about Veterans per se (*N* = 915)

Variable	N (%)
Subject discussed	
Individual Veteran(s)	394 (43.1)
Veterans as a group or not focused on individuals	521 (56.9)
Deployment	
Not applicable or not specified	596 (65.1)
Afghanistan	83 (9.1)
Korean War	10 (1.1)
World War II	144 (15.7)
Foreign peacekeeping	12 (1.3)
Home service	14 (1.5)
Multiple deployments	28 (3.1)
Other	28 (3.1)
Branch	
Not applicable or not specified	645 (70.5)
Canadian Army	153 (16.7)
Royal Canadian Navy	31 (3.4)
Royal Canadian Air Force	37 (4.0)
Other (e.g., foreign military)	49 (5.4)
Sex	
Not applicable or not specified	451 (49.3)
Male	390 (42.6)
Female	74 (8.1)

A more granular content analysis of news media content about Veterans is given below in Table 3. As can be seen, the most common underlying theme is honour or commemoration of Veterans which occurred in over half (56%) of the articles, particularly those in October and November. An example headline is “Lest we forget; City marks Remembrance Day with small ceremony, virtual audience”. Similarly, over half of the articles (59%) quote a Veteran or figure from a Veteran organization, and just under half (44%) quote an expert, official or leader.

**Table 3:** Themes and content of articles about Veterans per se (*N* = 915)

Variable	YES	
	N	%
Themes		
Honour or commemoration of Veterans	512	56.0
Recovery or reintegration	71	7.8
Shortage of resources or poor quality of services/care	130	14.2
Danger, violence, or criminality	129	14.1
Discusses health or social service/social welfare interventions	82	9.0
Quotes Veteran or figure from Veteran’s organization	547	59.8
Quotes expert, official or leader	408	44.6
Quotes family or close friend	174	19.0
Stigmatizing content or remarks created by the journalist	3	0.3
Stigmatizing content or remarks made by a third party	8	0.9
Is suicide a theme?	61	6.7
Is PTSD a theme?	93	10.2
Is another mental illness a theme?	62	6.8
Depression/anxiety	16	1.7
Substance misuse or substance use disorder	7	0.8
Multiple/other or general mental health	39	4.3

Of note, only 1% of articles contained stigmatizing content or remarks while only 14% focused on danger, violence or criminality (often related to Lionel Desmond). However, there were few articles about (i) Veteran reintegration or recovery (8%); (ii) health or social interventions (9%); and (iii) issues related to shortages of resources or poor quality of care (14%).

As can be seen in Table 3, suicide was a theme in only 6.7% of articles, while PTSD was a theme in only 10.2% of articles, with little discussion of depression or anxiety (despite their high prevalence among Veterans, as explained in the introduction [2]). It is worth noting that many articles covered more than one of the mental health categories, particularly those centred on the Lionel Desmond incident given that he died by suicide and was also diagnosed with PTSD. In total, 50 articles in our sample covered both PTSD and suicide, with 53.8% of all articles on PTSD also covering suicide, and 82.0% of all articles on suicide also covering PTSD. In other words, there was considerable overlap between the articles discussing PTSD and the articles discussing suicide, and most articles covering PTSD did so while simultaneously discussing suicide.

The sub-sample of articles discussing PTSD is presented in Table 4. As can be seen, danger, violence and criminality is a theme in over 60% of these articles, while less than 1 in 3 articles discuss recovery, reintegration or health/ social service interventions. Around half included a quote from a Veteran or an expert, while a handful contained stigmatizing comments. Stigmatizing comments were defined as “sensationalized language about veterans (e.g. ‘broken hero’, ‘ticking time-bomb’) or about veterans with mental illness (e.g. ‘nutcase’, ‘psycho’), or broad negative generalizations about veterans, such as implying that all veterans are inherently violent, unemployable or suicidal”. These few stigmatizing comments rarely came from journalists, but often involved quotes from others, for example a judge in a court case who linked pedophilia to military service and PTSD (example headline: “Military veteran spared jail because his pedophilia was triggered by PTSD: judge”).

**Table 4:** Content of articles about PTSD (*N* = 93)

Variable	YES	
	N	%
Is recovery or reintegration a significant theme?	28	30.1
Is shortage of resources or poor quality of services/care for Veterans a theme?	38	40.9
Is danger, violence, or criminality a theme?	58	62.4
Are health or social welfare interventions discussed in the text?	27	29.0
Are Veterans or figures from Veteran’s organizations quoted in the text either directly or paraphrased?	39	41.9
Are experts, officials, or community leaders quoted in the text either directly or paraphrased?	58	62.4
Are family relations or close friends to a Veteran quoted in the text either directly or paraphrased?	28	30.1
Does the story contain stigmatizing content or remarks created by the journalist?	2	2.2
Does the story contain stigmatizing content or remarks made by a third party?	6	6.5

A comparison of the sub-set of articles about PTSD (Table 4) with articles about Veterans per se (Table 3) demonstrates that articles about PTSD were much more likely to discuss danger, crime or violence (62% vs 14%), but also more likely to discuss recovery and reintegration (30% vs 8%), health and social welfare interventions (29% vs 9%) and shortages of resources and poor quality of services (41% vs 14%). This reveals a complex pattern, revisited in the discussion.

The content of the sub-set of articles focused on suicide is given in Table 5. Of note, 54 of these articles (89%) were focused on the Lionel Desmond incident, indicating that issues surrounding Veteran suicide were rarely covered in routine reporting, with only seven other stories about suicide in the course of the 12-month period.

**Table 5:** Content of articles about suicide (*N* = 61)

Variable	YES	
	N	%
Topic		
Suicide death	1	1.6%
Suicide attempt	0	0.0%
Murder-suicide	54	88.5%
Event/policy/research	1	1.6%
Other	5	8.2%
Headline includes the word ‘suicide’ or a synonym	14	23.0%
Mentions the suicide method used		
Yes, alludes to method	1	1.6%
Yes, passing direct mention	32	52.5%
Yes, in detail	8	13.1%
Mentions the suicide location	23	37.7%
Gives a monocausal explanation of suicide	0	0.0%
Glamourizes/romanticizes the death	0	0.0%
Includes sensational language	2	3.3%
Uses discouraged words/phrases such as ‘commit suicide’	1	1.6%
Provides help-seeking information such as helpline number	3	4.9%
Includes a quote by a suicide expert	31	50.8%
Includes a quote by a Veteran	17	27.9%
Includes a quote by the suicide bereaved	19	31.1%
Tries to educate the public about suicide and broader related issues	8	13.1%

In terms of adherence to Mindset suicide reporting guidelines, there was strong adherence to many recommendations. For instance, no articles gave a monocausal explanation of suicide, nor glamorized or romanticized the suicide. Similarly, virtually no articles used discouraged words, phrases or sensational language and suicide experts were quoted in over half of the articles. However, there was low adherence to other recommendations. Less than 5% provided help-seeking information, only 13% tried to educate the public about suicide and broader issues, over 50% mentioned the suicide method used, and more than 1 in 3 mentioned the suicide location.

## Discussion

The results reveal some encouraging findings as well as room for improvement regarding media coverage of Canadian Veterans. As stated in the introduction, an amassed corpus of US research indicates that the US media often focuses on factors such as trauma, emotional instability and drugs/alcohol when reporting Veterans, with a lack of positive stories and an emphasis on Veterans as victims [14–17]. However, the present study indicates that the Canadian media typically represent Veterans in a positive manner, with a focus on commemoration and honour in over 50% of articles, often in reference to World War 2. Similarly, there is very little discussion of drug/alcohol abuse in the Canadian articles, and virtually no instances where journalists use stigmatizing language.

Moreover, the articles about PTSD and suicide generally follow best practice guidelines, with high levels of adherence to the Mindset recommendations. This is consistent with other Canadian research on generic media coverage of mental illness and suicide, which shows that the Canadian media has significantly improved its coverage of these issues in recent years, with better adherence to core guidelines than the US media [27, 45, 50].

For example, the present study found that only 3% of articles about Veteran suicide included sensational language, 0 % attributed suicide to a single cause, 23% included ‘suicide’ in the headline, while 51% included a quote by a suicide expert, and 5% included help-seeking information. In contrast, a recent US study of media coverage of Veteran suicide found that 60% of articles included sensational/dramatic language, 60% attributed Veteran suicide to a single cause and 40% included ‘suicide’ in the headline, while 0 % contained help-seeking information or quoted suicide experts [22]. That said, the present paper reveals five issues regarding the pattern of reporting of Veterans in the Canadian media that may require further attention and action.

First, articles about Veterans per se rarely discuss recently-discharged Veterans, except in the context of highly-anomalous cases such as Lionel Desmond and Jonathan Vance. Indeed, less than 10% of articles focused on Afghanistan Veterans, and many of these focused on Lionel Desmond. This is concerning, given that over 40,000 Canadian Forces personnel served in the Afghanistan theatre. Indeed, research indicates that many such Veterans have faced specific challenges making the transition to civilian life [28], with some studies indicating that these Veterans are at high risk of suicide and social isolation [3, 4]. Greater and more diverse media coverage of Afghanistan Veterans may shine a spotlight on their specific issues, which in turn may catalyze positive social and institutional change. Moreover, some other Veterans are rarely featured in the media, including female Veterans, Royal Canadian Navy Veterans, and Royal Canadian Air Force Veterans. Again, this can lead to wider public ignorance about issues faced by such Veterans, and more holistic coverage may be beneficial.

Second, the study indicates that articles about Veterans tend to cluster around key events during the course of a year, with Veterans remaining relatively invisible outside of these clusters. The first cluster appeared in the weeks surrounding Remembrance Day (November 11), accounting for over 50% of articles. The second cluster appeared in February/ March 2021, consisting of two sub-clusters (i) the inquest into the highly anomalous but newsworthy Lionel Desmond murder-suicide, and (ii) the sexual misconduct allegations leveled against military Veterans, especially General Jonathan Vance. Of note, the first cluster could be framed as a ‘positive cluster’, as the weeks around Remembrance Day typically illuminate Veterans’ sacrifices and their ongoing contribution to society. However, the two sub-clusters in 2021 could be framed as ‘negative clusters’, as they focus on adverse phenomena including murder-suicide and sexual misconduct. Such a focus could skew wider public perceptions, leading readers to associate Veterans with behaviours and attitudes that are harmful to self and others. This suggests the importance of fair, balanced and accurate coverage of Veterans throughout the year, with the inclusion of positive, hopeful and holistic stories of reintegration and recovery.

Third, with regards to PTSD, over 60% focused on danger, crime or violence (with most of these related to the Lionel Desmond inquest); while only around 1 in 3 focused on recovery, rehabilitation, or health/social service intervention. Moreover, the majority of articles about PTSD simultaneously discussed some aspect of suicide. These results overlap with studies elsewhere indicating that media coverage of PTSD often focuses on crime and violence, with fewer articles on recovery or rehabilitation [18–21]. Such common content could suggest to the reader that PTSD is associated with crime, violence and suicide, and that it is a disorder with low recovery rates and few evidence-based interventions. In fact, there are numerous efficacious treatments for PTSD, and high recovery rates when given the right services and supports [57].

Again, this implies the need to diversify coverage of PTSD in the Canadian media so that it includes a greater proportion of stories containing themes of hope, recovery and successful mastery of challenging situations. Indeed, inclusion of such themes may foster a Papageno effect (discussed in the introduction) which has been shown in various studies to mitigate suicidality in susceptible individuals, including those with pre-existing mental health issues such as PTSD [34, 39, 47, 48].

That said, it is important to note that only 2.2% of articles about PTSD in the present study contained stigmatizing content created by the journalist, meaning that articles did not state or intimate that all Veterans are violent criminals of whom the public should be frightened. In fact, a qualitative content analysis of media articles in the immediate months following the actual Lionel Desmond murder-suicide in 2017 found that journalists typically wrote about this incident in compassionate terms, calling for more action to help Veterans’ mental health, as well as more support for Veterans who are in transition to civilian life [58]. In other words, content in articles about PTSD is typically high-quality, even when reporting crime, violence and suicide; but the disproportionate focus on crime, violence and suicide is an issue in itself, as discussed below.

Fourth, there remains room for improvement in the reporting of Veteran suicide. As stated, articles about suicide rarely include putatively-protective information such as a help-line number or other help-seeking resources, and few try to educate the public about suicide. More effort could be made to include such information in the future. Moreover, it is important to repeat that 54 of 61 articles (89%) covering the topic of Veteran suicide relate to the Lionel Desmond murder-suicide. On the one hand, it is worth noting that several studies from Canada and elsewhere (discussed in the introduction) indicate that reporting of murder-suicides is associated with lower subsequent suicide rates [38–40]. This is because susceptible individuals are unlikely to identify with a public ‘villain’ who has incurred lower social status by their violent actions, meaning that this pattern of reporting will be unlikely to contribute to any Werther effect in Canadian Veterans.

On the other hand, this focus on a single anomalous case essentially means that almost all suicide-related articles about Veterans were about a specific serious crime, which is a major finding in itself. This near exclusive focus on a single outlier means that there are very few generic articles about suicide prevention, helpful resources and modifiable risk factors in relation to Veteran suicide. For example, a recent report (discussed in the introduction) notes that 17% of Veterans have been diagnosed with a mood disorder [2], which is one of the most significant risk factors for suicidality in Canadian Veterans [59, 60]. However only 1.7% of all media articles about Veterans in the present study had depression or anxiety as a theme. In other words, there is a need to further diversify coverage of suicide and Canadian Veterans, with more discussion of suicide prevention, helpful resources, modifiable risk factors and broader social issues.

Fifth, articles about suicide and PTSD tended to adhere to Mindset guidelines, which is overall a positive finding. However, it may be the case that the Mindset guidelines are weaker than other similar guidelines inasmuch as they may be missing important advice for journalists. For example, the Mindset suicide guidelines do not include explicit bullet-points advising journalists to cover hopeful stories of resilience and mastery of crises. These are arguably the most important stories for suicide prevention and are instrumental in fostering a Papageno effect [34, 39, 47, 48].

That said, the newest edition of the Mindset guidelines (published after the present study began) does include new bullet-points such as ‘do present suicide as mainly arising from treatable mental illness, thus preventable’ which is a positive step, but Mindset still may not completely capture the gamut of key variables relevant to suicide prevention. In other words, the findings indicate high adherence to Mindset guidelines, but this does not necessarily mean that media coverage is optimal, and there remains room for improvement in coverage of PTSD and suicide issues related to Veterans in Canada. In some respects, the issue is not what is being said by the media with regards to Veteran PTSD and suicide, but what is not being said.

There are several limitations to this study. The first limitation is that it only included articles from print or online media, and did not include other media such as television, radio, or social media. Analysis of these other media (especially social media) may have revealed different patterns. Second, the study period spanned only 12 months, which can only give a snapshot of current portrayals, but can not give long-term information about time-trends. As such, longitudinal research is necessary to assess change over time. Third, the study only collected data from a single jurisdiction (Canada), and while comparisons have been made with studies from other jurisdictions, these other studies employed different methodologies and occurred during different time frames. Purpose-built between-country comparative studies would provide a better foundation for the comparison of media coverage between different jurisdictions. Fourth, data collection occurred during the COVID-19 pandemic, which may have had an influence on reporting patterns, meaning that the July 2020–July 2021 time period is not a ‘typical’ year. Fifth, we did not engage in correlational analysis between some of the key variables, nor make any comparisons in coverage between types of media (e.g. English-language versus French-language, page one stories versus inside pages stories etc.), as this was beyond the scope of the present study, and such an approach would be hampered by small cell sizes and low number of cases among relevant variables. The article is purposely descriptive to give an overview of general patterns and adherence to guidelines, which is consistent with the pointed aims of the study and addresses a gap in the literature on Veteran portrayals in the Canadian media. These limitations could be addressed in future purpose-driven research with larger samples conducted over a multi-year period.

## Conclusions

To conclude, the present study reveals that the Canadian news media frequently focus on issues of honour and commemoration when reporting Veterans. Compared to the US media, there are far fewer articles on Veterans as victims or drug/alcohol issues in Veterans. Moreover, articles about suicide and PTSD tend to adhere to Mindset guidelines, though there remains room for improvement when reporting these issues. However, there are also very few articles about Afghanistan Veterans, female Veterans, and Veterans who served outside the Canadian army.

In sum, the results suggest a need for more educational outreach to help Canadian journalists responsibly report Veterans and their issues, with a focus on (i) diversifying coverage to represent the full heterogeneity of Veterans and Veteran experience; (ii) improved adherence to Mindset guidelines when reporting suicide and PTSD in Veterans; (iii) including more stories focusing on mastery of crises, resilience in the face of adversity and messages of hope and recovery in relation to PTSD and suicide; and (iv) reporting Veterans and their experiences outside of newsworthy clusters and anomalous events, especially focusing on successful reintegration and life progress. Such efforts may help shape a wider social climate of respect, inclusion and support for all Canadian Veterans that may lead to less stigma and stereotypes. This can facilitate Veteran integration into society, which in turn can promote their mental health and well-being, and may ultimately help prevent suicide and foster recovery from PTSD and other mental illnesses.

## Supplementary Information

**Additional file 1.** News sources used, by media type and scope. News sources used in this examination, organized by media type and scope.

## Data Availability

The raw data for this study consists of newspaper articles. Many of these articles are behind a paywall on the news media websites listed in Additional file 1, or only available via paid subscription to a database. Hence, we do not have the right to make these newspaper articles publicly available, as they were obtained through paid subscription to Factiva software in the current study. However, a list of the 915 coded articles is available from the authors on reasonable request, which can be used to obtain the raw data behind the paywalls.

## Abbreviation

PTSD: Post Traumatic Stress Disorder
