# Supplementary material for: Media coverage of Canadian Veterans, with a focus on post traumatic stress disorder and suicide
Source: BMC Psychiatry. 2022 May 16;22:339. doi: 10.1186/s12888-022-03954-8 (PMC9109435; doi:10.1186/s12888-022-03954-8)
Supplement: Supplementary file 1 — Additional file 1. News sources used, by media type and scope. News sources used in this examination, organized by media type and scope. [file 12888_2022_3954_MOESM1_ESM.pdf]

**On-line News**	**Regional**	**(by circulation)**
1. CBC.CA News	1. Toronto Star	21. Regina Leader-Post
2. Global News	2. Toronto Sun	22. The Telegram
3. CTV National News	3. Vancouver Sun	23. Thunder Bay Source[1]
4. CTV News (Toronto, Windsor)	4. Vancouver Province	24. Kingston Whig Standard
5. HuffPost Canada	5. Hamilton Spectator	25. Lethbridge Sun Times[1]
6. Macleans Online	6. Calgary Herald	26. Brantford Expositor
7. La Presse.ca	**7.** Winnipeg Free Press	27. St Catharines Standard
8. La Tribune	8. Edmonton Journal	28. Peterborough Examiner
9. Le Devoir	9. Ottawa Citizen	29. The Guardian
10. Le Droit	10. Chronicle-Herald	30. Owen Sound Sun Times
11. Le Soleil	11. Montreal Gazette	31. Barrie Examiner
12. Métro Montréal	12. London Free Press	32. Niagara Falls Review
13. Radio-Canada	13. Victoria Times Colonist	33. Guelph Mercury
14. TVA Nouvelles	14. Winnipeg Sun	34. Kelowna Capital News[1]
	15. Waterloo Region Record	35. Prince George Citizen
	16. Calgary Sun	36. Welland Tribune
**National**	17. Windsor Star	37. Sudbury Star
1. Globe and Mail	18. Edmonton Sun	38. Sault Star
2. National Post	19. Ottawa Sun	39. 24 Heures Montréal
3. StarMetro	20. Saskatoon StarPhoenix	40. Journal de Montréal
		41. Journal de Québec
